# SFTS phlebovirus promotes LC3-II accumulation and nonstructural protein of SFTS phlebovirus co-localizes with autophagy proteins

**DOI:** 10.1038/s41598-018-23610-0

**Published:** 2018-03-27

**Authors:** Yue Sun, Miao-miao Liu, Xiao-ying Lei, Xue-jie Yu

**Affiliations:** 1 School of Public Health, Shandong University, Jinan City, Shandong Province China; 2 School of Public Health, Jining Medical University, Jinan City, Shandong Province China; 3 School of Health Sciences, Wuhan University, Wuhan City, Hubei Province China

## Abstract

Autophagy is essential for eukaryotic cell homeostasis and can perform both anti-viral and pro-viral roles depending on the kinds of viruses, cell types and cell environment. Severe fever with thrombocytopenia syndrome phlebovirus (SFTSV) is a newly discovered tick-borne virus in the *Phenuiviridae* family that causes a severe hemorrhagic fever disease in East Asia. In this study we determined interactions between SFTSV and autophagy. Our results showed that LC3-II (microtubule associated protein 1 light chain 3-II) protein accumulated from 4 h to 24 h after SFTSV infection compared to mock-infected Vero cells, and the use of E64d and pepstatin A did not affect the expression of LC3-II protein, which indicated that the increased LC3-II may be the result of inhibition of autophagic degradation caused by SFTSV infection. However, knockdown of *LC3B* promotes SFTSV replication, which indicated a negative role of LC3B protein in SFTSV replication. We also detected co-localization of SFTSV non-structure (NSs) protein with LC3B, p62 and Lamp2b respectively in SFTSV infected Vero cells, which indicated the possibility of selective autophagy or chaperone-mediated autophagy involving in SFTSV infection. Our results indicated that SFTSV infection promotes LC3 accumulation and several proteins of the autophagy pathway co-localize with NSs protein during SFTSV infection.

## Introduction

Severe fever with thrombocytopenia syndrome (SFTS) is an emerging hemorrhagic fever disease, which was first reported in 2010 in China and subsequently reported in South Korea and Japan^1–3^. The major clinical symptoms of SFTS include fever, thrombocytopenia, gastrointestinal symptoms and leukopenia with a fatality rate of 12–50%^1,2^. SFTS is caused by a tick-borne virus, severe fever with thrombocytopenia syndrome phlebovirus (SFTSV)^1,4–6^, which can also be transmitted between humans occasionally^7,8^. SFTSV is a member of the *Phenuiviridae* family in the order *Bunyavirales*, which was recently created by the International Committee on Taxonomy of Viruses^9^.

SFTSV is a single-stranded negative-sense RNA virus with 3 genomic segments, L, M, and S^1^. The L and M segments encode viral RNA polymerase and viral envelope glycoproteins, respectively. The S segment is an ambisense RNA encoding the nucleoprotein (NP) and the nonstructural protein (NSs)^10^. The NSs proteins of various viruses from the *Phenuiviridae* family and other families of *Bunyavirales*^9^ order have been reported to have important effects on interacting with host responses through different mechanisms^11^. For example, NSs protein of Rift Valley fever virus (RVFV) forms fibrillar in the nuclei of infected cells, targets cellular TFIIH transcription factor and blocks interferon production^12–16^. The NSs protein of SFTSV has been reported to play essential roles in SFTSV replication and host responses^17^. Previous studies have reported that the NSs protein of SFTSV suppressed the beta interferon (IFN-β) and NF-κB promoter activities^18^. SFTSV NSs can suppress the exogenous Type I IFN-induced Jak/STAT, which may be one of the mechanisms of SFTSV evasion of host immune surveillance^17^. NSs protein can form viroplasm-like structures (VLSs) in infected and transfected cells. The NSs-formed VLS may be implicated in the replication of SFTSV^11^. NSs protein interacts and co-localizes with RIG-I, the E3 ubiquitin ligase TRIM25, and TANK-binding kinase 1 (TBK1) into SFTSV NSs-induced cytoplasmic structures, through which it may inhibit IFN responses^19^.

Autophagy is not only a process of cellular homeostasis and stress response, but also has anti-viral and pro-viral roles. Viruses can break, escape from or even take advantage of autophagy for their replication^20^.

Some viruses have been reported to interfere with autophagy. For instance, the Herpes Simplex Virus 1 protein ICP34.5, the Kaposi’s Sarcoma herpesvirus (KSHV) orf16 protein and the MHV-68 M11 protein can all bind to Beclin 1 to inhibit the formation of autophagosomes and autolysosomal degradation in neurons^21–24^; Coxsackievirus B3 (CVB3) and poliovirus inhibit autophagy by blocking autophagosome maturation or degradation^25,26^. The viral proteins Nef and M2 of human immunodeficiency virus-1 (HIV-1) and influenza A virus (FluAv) block autophagosome maturation by a still poorly defined mechanism that depends on their interactions with Beclin-1^27,28^.

Autophagy has divergent roles in viral infection^29^. The autophagosome can selectively target virions and virus components for degradation via the autolysosome, such as the recognition of Sindbis virus capsid by p62^30,31^. Furthermore, the autophagosome can specifically deliver intracellular pathogen-associated molecular patterns (PAMPs) to endosomal pattern recognition receptors (PRRs) and MHC-loading compartments to initiate innate and adaptive immune responses such as the VSV (Vesicular Stomatitis Virus) and CVB3, which deliver viral replication intermediates to TLR7 and TLR3, respectively^32–34^. Autophagy can also enhance viral replication through various pathways. For example, CVB3 and enterovirus 71 (EV71) can induce the double-membrane vesicles (DMV) that are characteristic of autophagosome-like structures, and autophagosomes are dispensable for biogenesis of viral replication sites^26,35–38^. The nonstructural protein 1 (NS1) of Japanese encephalitis virus (JEV) co-localizes with endogenous LC3 (microtubule associated protein 1 light chain 3) and EDEM1 (ER degradation enhancer, mannosidase α-like 1) to promote JEV replication^39^. Until now the mechanism of SFTSV interacting with autophagy is not clear. In this study we have explored the interaction between SFTSV and autophagy in Vero cells.

## Results

### LC3-II protein was accumulated in SFTSV infected cells

**Figure 1:**
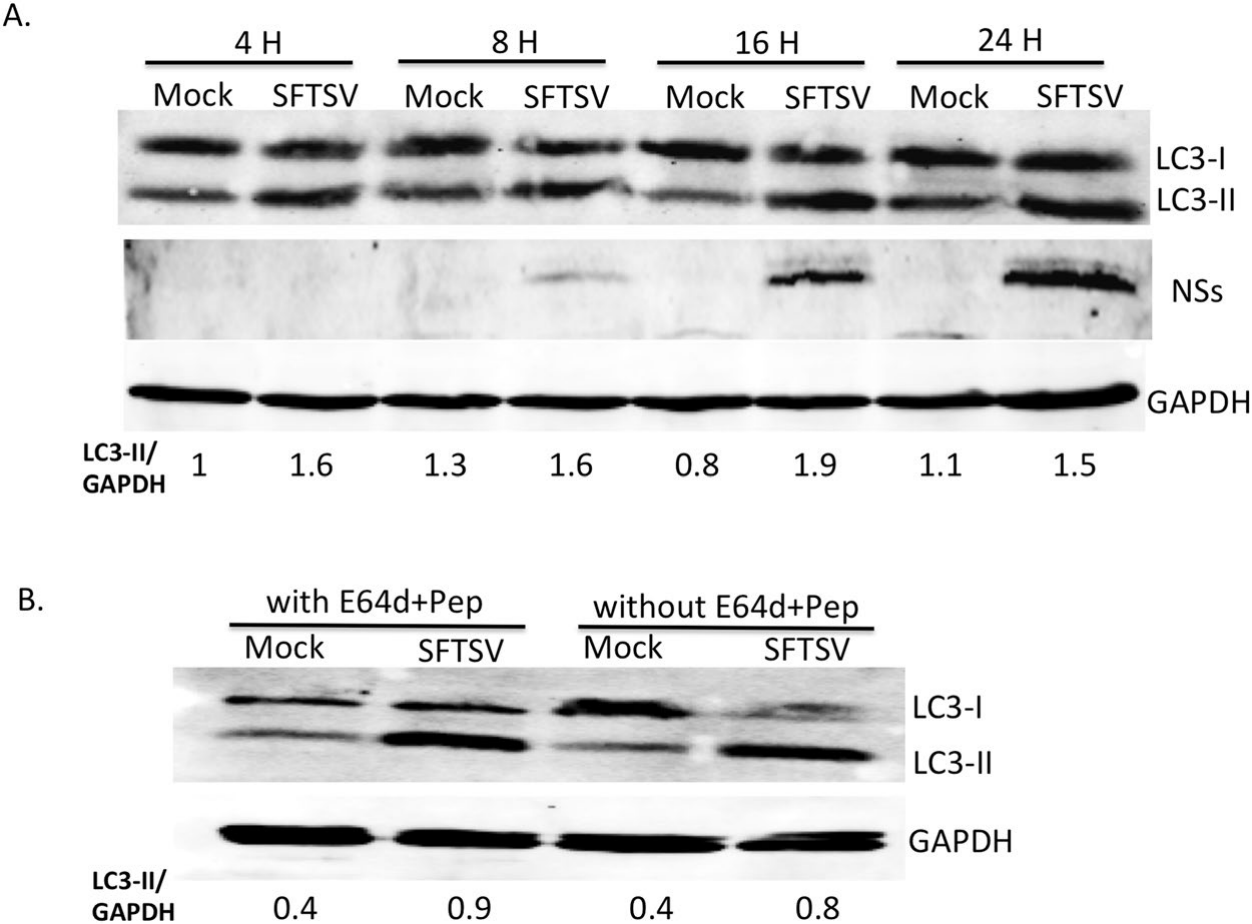
LC3-II protein was accumulated in SFTSV infected cells. (**A**) The band of LC3-II protein in SFTSV-infected cells is darker than that in mock-infected cells. All antibodies were reacted with the same membrane. (**B**) There is no significant difference of LC3-II band between infected cells with inhibitors (0.9) and infected cells without inhibitors (0.8).

To detect how SFTSV affects autophagy *in vitro*, we first detected LC3B protein expression at different time points after SFTSV infection by Western blot. Our results showed that LC3-II protein accumulated in SFTSV infected Vero cells compared to the mock-infected cells (Fig. 1A), which indicated that SFTSV infection might promote LC3-II synthesis or inhibit autophagic degradation.

E64d and pepstatin A inhibit lysosomal proteases and interfere with autolysosomal digestion^40^. To precisely interpret the mechanism of accumulation of LC3-II by SFTSV infection, we further investigated whether E64d and pepstatin A affected LC3-II expression in SFTSV-infected Vero cells. Our results showed that under SFTSV infection, there is no significant difference of LC3-II expression between inhibitor-treated and non-inhibitor-treated cells (Fig. 1B). This indicated that the increased LC3-II might be result of inhibition of autophagic degradation by SFTSV infection.

### SFTSV infection induced formation of LC3-positive structures

**Figure 2:**
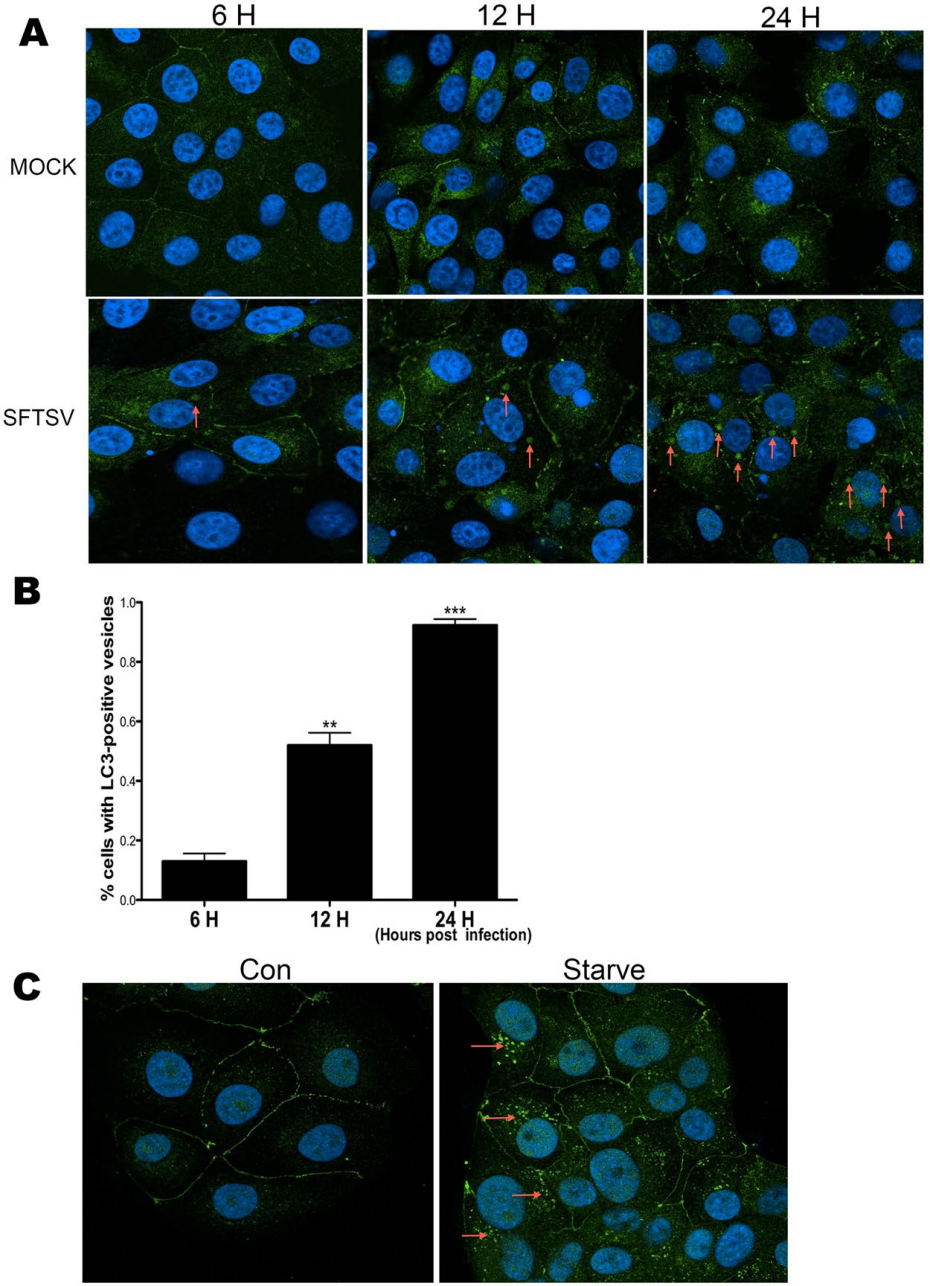
SFTSV infection leads to formation of LC3-positive structures (**A**) LC3-positive structures can be seen in SFTSV-infected cells (red arrows). (**B**) The percentage of cells with LC3-positive structures. Bars represent the standard error. Mean ± SEM were: 6 h: 0.13 ± 0.03, n = 3; 12 h: 0.52 ± 0.04, n = 3, p: 0.0014; 24 h: 0.92 ± 0.02, n = 3, p < 0.0001. T-test was used to determine statistical significance versus 6 h. At least three pictures were analyzed at each time point and experiments were repeated for three times, showing consistent results. (**C**) Typical LC3 puncta could be seen in starved cells.

To further investigate how autophagy was affected by SFTSV infection, we next detected LC3 expression in both SFTSV-infected and mock-infected cells by confocal microscopy. We detected LC3 expression at different time points after SFTSV infection. Our results showed that in mock-infected cells, LC3 was diffusely distributed at all times; while in SFTSV-infected cells, LC3 protein was clustered together as certain structures (Fig. 2A). Each SFTSV infected Vero cell contained approximately 1 to 3 LC3 positive structures, which were not observed in mock-infected cells. Furthermore, as the infection time was prolonged, the number of SFTSV-infected cells that contained LC3 positive structures increased (Fig. 2B). Then we detected LC3 protein in starved Vero cells by confocal microscopy, which showed the typical LC3 puncta (Fig. 2C). We found that the LC3-structures in SFTSV infected Vero cells were different from the punctate LC3 pattern in starved cells where typical autophagy occurred. This suggested that the increased LC3-II in SFTSV infected cells might be LC3-positive structures instead of autophagosomes, which indicates that SFTSV infection has induced the formation of LC3-positive structures that are different from LC3 puncta formed in typical autophagy process.

### LC3B inhibited SFTSV replication

**Figure 3:**
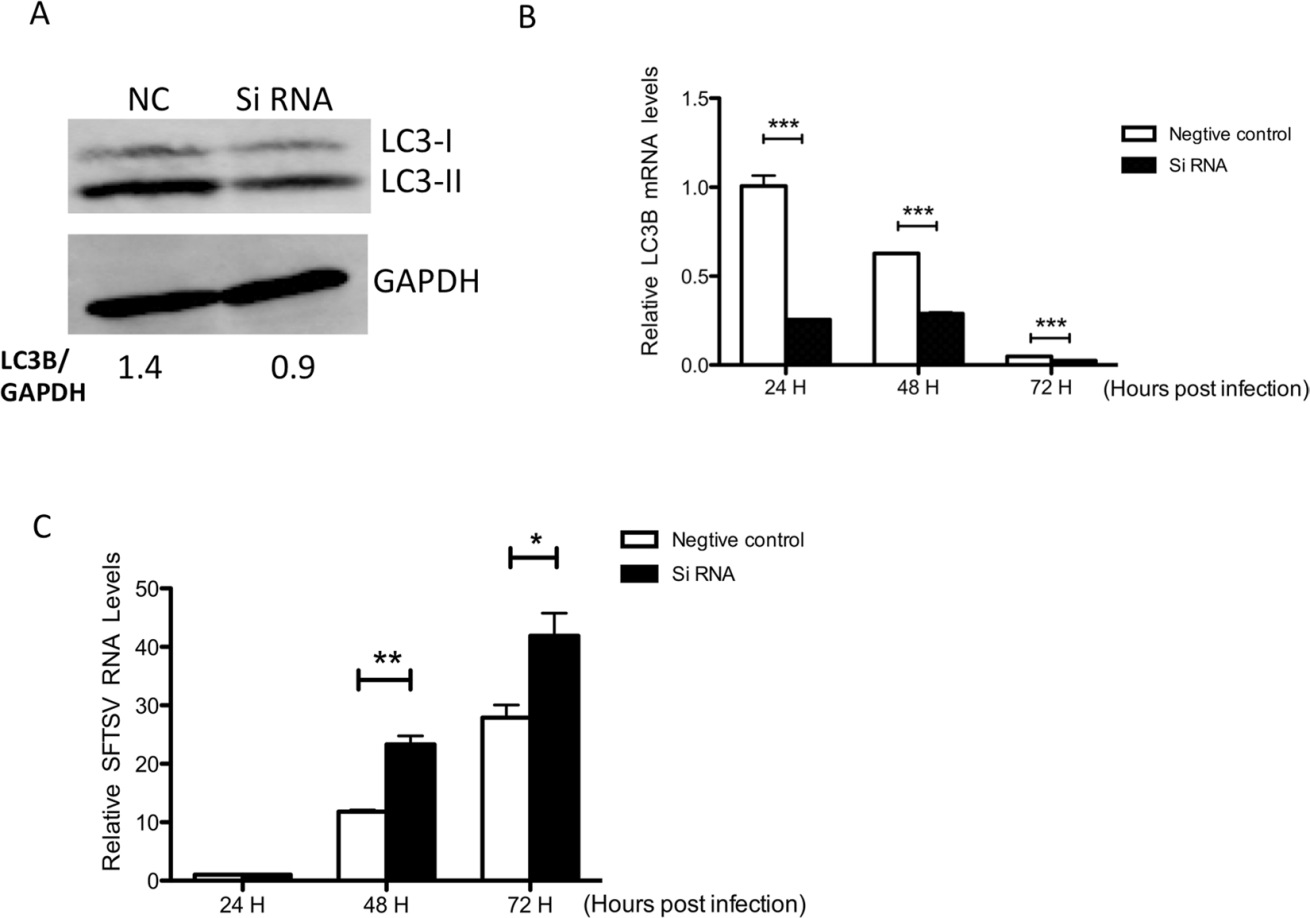
LC3B inhibits SFTSV replication. (**A**) LC3B protein expression decreased in siRNA-transfected group. (**B**) *LC3B* mRNA expression decreased. Mean ± SEM were: 24 h (NC): 1.00 ± 0.06, n = 3, 24 h (SiRNA): 0.26 ± 0.01, n = 3, p = 0.0002; 48 h (NC): 0.63 ± 0.01, n = 3, 48 h (SiRNA): 0.29 ± 0.01, n = 3, p < 0.0001; 72 h (NC): 0.05 ± 0.001, n = 3, 72 h (SiRNA): 0.02, n = 3, p < 0.0001. (**C**) The SFTSV RNA level decreased over time compared to the control group. Mean ± SEM were: 48 h (NC): 11.80 ± 0.28, n = 3, 48 h (SiRNA): 23.30 ± 1.46, n = 3, p = 0.0015; 72 h (NC): 27.89 ± 2.17, n = 3, 72 h (SiRNA): 41.90 ± 3.87, n = 3, p = 0.0343. Bars represent the standard error. T-test was used to determine statistical significance between negative control group and siRNA group at each time point respectively. Experiment of each group was repeated for three times, showing consistent results.

To further confirm the effect of LC3 on SFTSV infection, we performed knockdown of *LC3B* gene in Vero cells by RNA interference. *LC3B*-depressed cells showed lower expression of LC3B protein, which proved the RNA interference to be effective (Fig. 3A and B). Then we detected the RNA levels of SFTSV in supernatant of cells respectively from LC3B-depressed group and control group at 24 h, 48 h and 72 h post infection by real-time PCR assay. Results showed that the SFTSV RNA levels increased significantly in the supernatant of LC3B-downregulated cells compared to that of the control group (Fig. 3C). This suggests that LC3B protein plays a negative role in SFTSV replication.

### NSs protein positive structures were found in the SFTSV-infected cells

We used antibodies against NSs protein to react with SFTSV-infected cells and found remarkable high-density and round structures existed in the infected cells.

**Figure 4:**
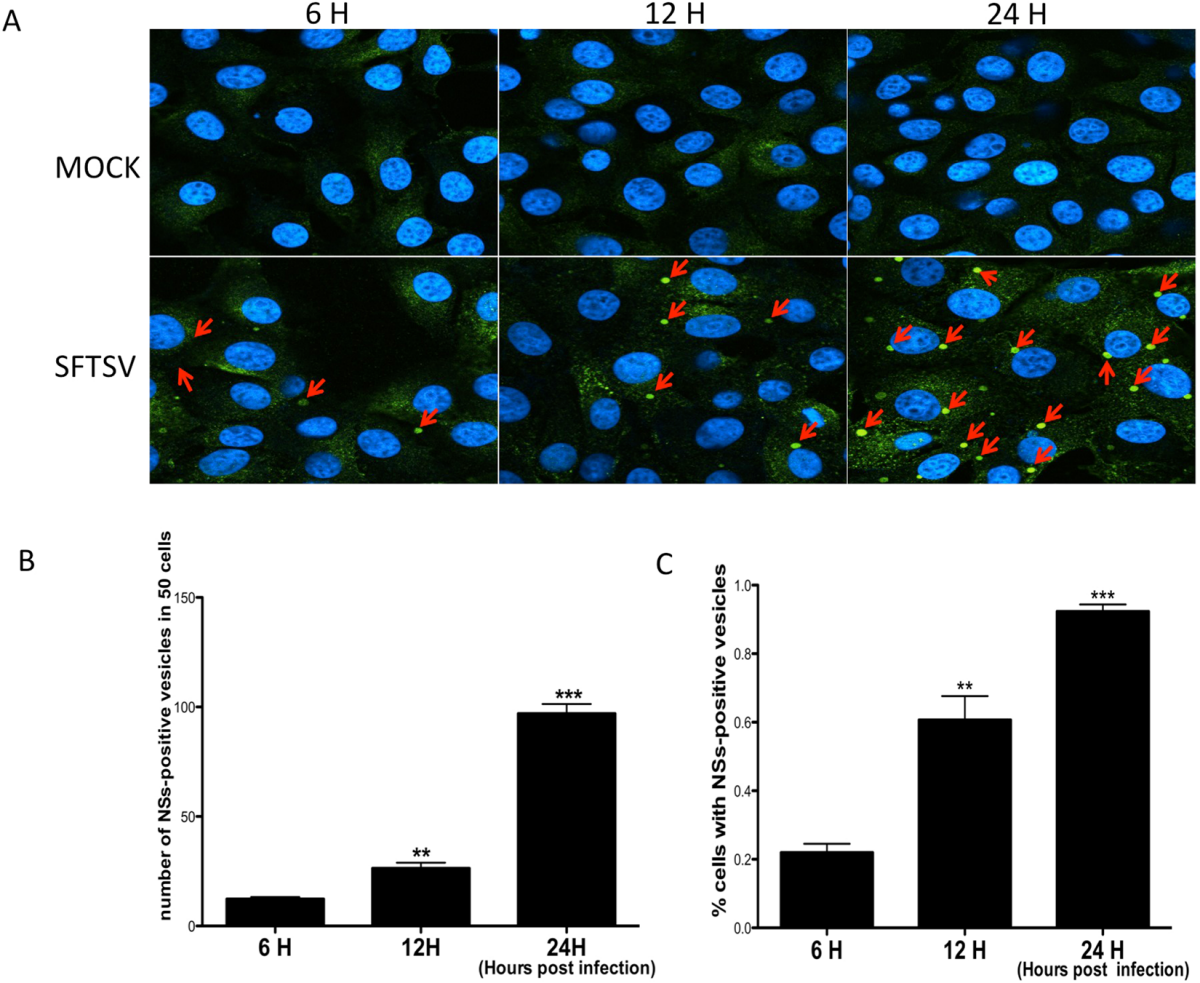
NSs protein positive structures are found in the SFTSV-infected cells. (**A**) There were obviously NSs-positive structures in SFTSV-infected cells (red arrows) while none could be seen in control group. (**B**) The NSs-positive structures were counted in 50 cells in each experiment. Mean ± SEM were: 6 h: 12.33 ± 0.89, n = 3; 12 h: 26.33 ± 2.60, n = 3, p: 0.007; 24 h: 97.00 ± 4.36, n = 3, p < 0.0001. (**C**) The percentage of cells with NSs-positive structures. Mean ± SEM were: 6 h: 0.22 ± 0.03, n = 3; 12 h: 0.61 ± 0.07, n = 3, p: 0.0063; 24 h: 0.92 ± 0.02, n = 3, p < 0.0001. T-test was used to determine statistical significance versus 6 h. At least three pictures were analyzed at each time point and experiments were repeated for three times, showing consistent results.

Generally each SFTSV-infected cell contained 2 to 3 structures, which were not found in mock-infected cells. These structures existed only in the cytoplasm and not in the nuclei of infected cells. We tested cells at different time points post infection and found that both the number and density of these structures in infected Vero cells increased over time (Fig. 4A and B). As the percentage of cells that contain NSs-positive structures increased (Fig. 4C), nearly all infected cells could be found with these structures at 24 h after infection. Thus NSs-positive structures were found in SFTSV-infected cells, which were most likely the NSs-induced viroplasm-like structures (VLSs) as described previously^11^.

### NSs protein of SFTSV co-localized with LC3

**Figure 5:**
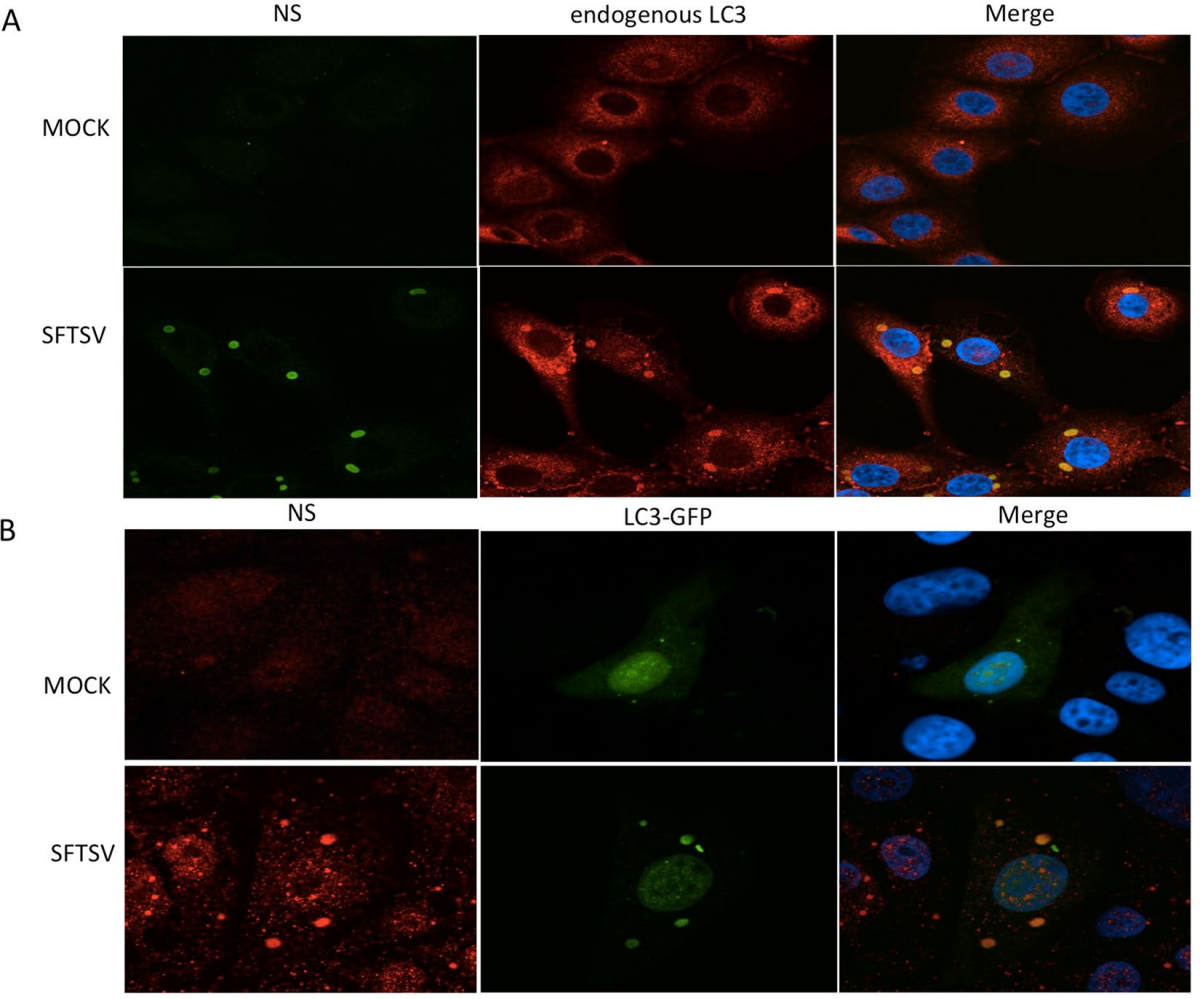
NSs protein of SFTSV co-localizes with LC3. (**A**) In SFTSV-infected cells, the NSs protein co-localized with LC3 protein in NSs-positive structures. (**B**) In SFTSV-infected cells, the EGFP-LC3B congregated into special structures and co-localized with NSs protein.

To further explore the relationship between NSs protein of SFTSV and autophagy, we detected the localization of NSs protein and LC3 simultaneously and found that in SFTSV-infected cells there is a remarkable overlap of unique structures between the NSs and endogenous LC3 (Fig. 5A). To further confirm the co-localization of NSs protein and LC3 protein, we transfected Vero cells with EGFP-LC3 and then infected them with SFTSV. In contrast with the control group, the infected cells had unique structures where the LC3-GFP and NSs protein presented a complete co-localization (Fig. 5B). This suggests that SFTSV infection induces the co-localization between NSs protein and the LC3 protein.

### NSs protein alone did not co-localize with LC3

**Figure 6:**
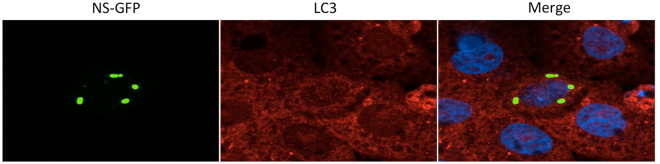
NSs protein alone is unable to co-localize with LC3. NSs-GFP protein congregated into typical structures, which were similar to structures that NSs protein formed in SFTSV-infected cells. However no co-localization was found between LC3 and NSs-GFP.

To determine whether NSs protein alone could co-localize with LC3 protein, we transfected the NSs-GFP plasmids into Vero cells and observed them with confocal microscopy. We found that no NSs-GFP protein co-localized with LC3 (Fig. 6), suggesting that NSs protein alone is unable to co-localize with LC3 or GFP fusion protein interfered with the co-localization of NSs protein with LC3.

### NSs protein co-localized with p62 and Lamp2b protein

**Figure 7:**
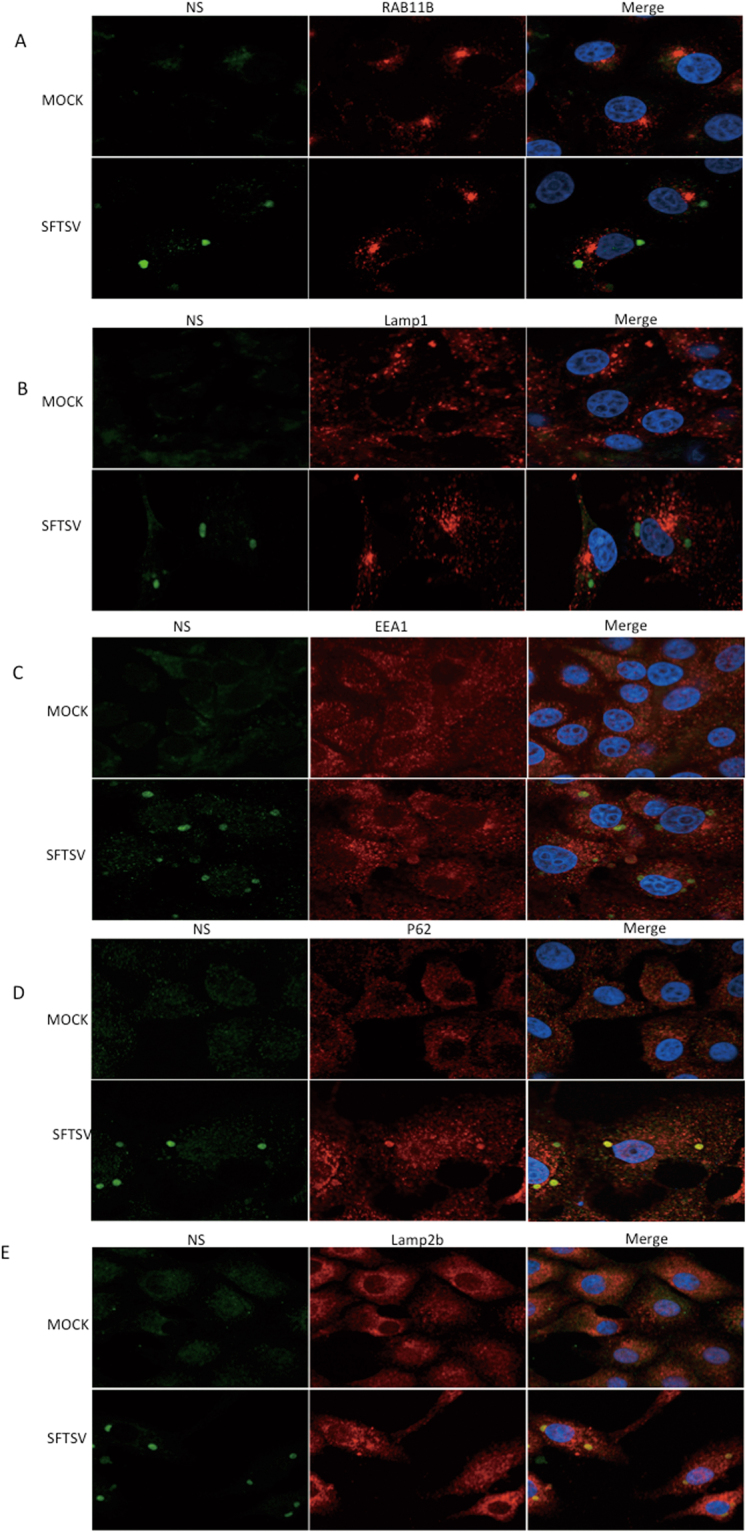
NSs protein co-localized with p62 and Lamp2b protein. (**A**–**E**) NSs-positive structures can be seen in SFTSV-infected cells; no co-localization was seen between Rab11b and NSs, Lamp1 and NSs, EEA1 and NSs. However obvious co-localizations were seen between NSs and p62, NSs and Lamp2b.

We further investigated how NSs proteins of SFTSV affected autophagy or other membrane metabolism pathways in cells during infection. We detected membrane related proteins including p62 (also called SQSTM1), Lamp1 (lysosome-associated membrane protein 1), Lamp2b (lysosome-associated membrane protein 2b), EEA1 (the early endosome antigen-1) and Rab11b in SFTSV-infected cells with confocal microscopy. We did not observe co-localization between NSs and Lamp1, EEA1 or Rab11b. These results suggested that NSs might not interact with these proteins directly (Fig. 7A,B and C).

P62 is a protein marker of autophagy and it serves as a link between LC3 and ubiquitinated substrates^41,42^. Lamp2b is a protein marker of lysosomes or autolysosomes^43^. We found that besides LC3, NS protein also co-localized with p62 and Lamp2b respectively (Fig. 7D and E), which further indicated that autophagy might play important roles in SFTSV replication.

### NSs protein alone could not lead to p62 or Lamp2b sequestration

**Figure 8:**
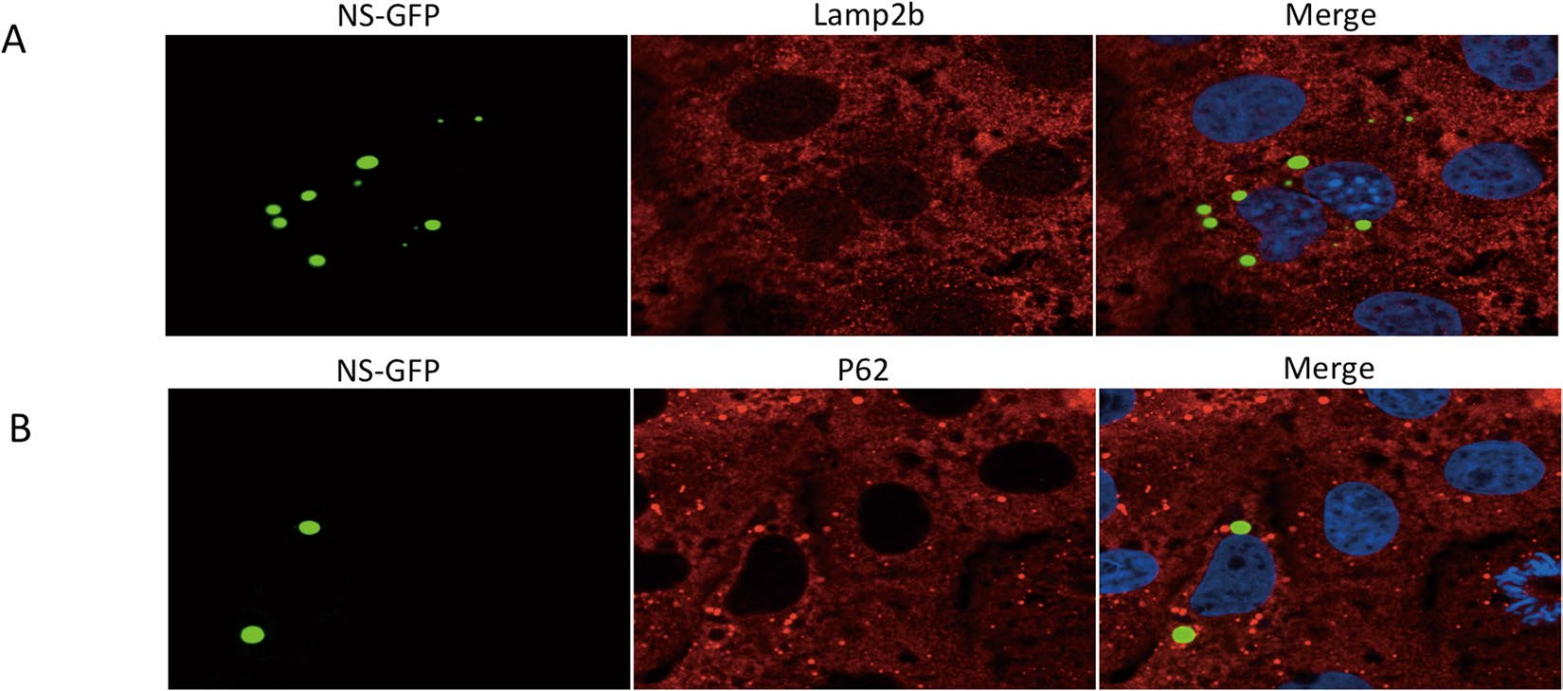
NSs protein alone could not lead to p62 or Lamp2b sequestration. (**A**,**B**) NSs-GFP protein congregated into typical structures, which were similar to structures that NSs protein formed in SFTSV-infected cells. However no co-localization was found between p62 and NSs-GFP, or between Lamp2b and NSs-GFP.

To further detect whether NSs protein alone could lead to sequestration of p62 or Lamp2b, we transfected the NSs-GFP vector into Vero cells. We did not observe the co-localization of NSs-GFP with p62 or Lamp2b (Fig. 8A and B). The results suggested that co-localization of NSs with P62 and Lamp2b is caused by SFTSV infection, while NSs protein alone is unable to sequester p62 or Lamp2b.

## Discussion

In this study we determined how SFTSV infection affected autophagy and how autophagy influenced SFTSV replication. We demonstrated that SFTSV could promote LC3-II accumulation. Down-regulated expression of LC3 protein led to SFTSV RNA up-regulation. We also demonstrated that the NSs protein of SFTSV could co-localize with LC3, P62 and Lamp2b proteins in SFTSV infected cells. However, the co-localization phenomenon of NSs with LC3, P62 and Lamp2b proteins disappeared in the NSs-GFP transfected cells.

Previous studies reported the activation of autophagy upon virus infection as inferred from the increased number of autophagic structures and the monitoring of LC3-I to LC3-II conversion in infected cells^44–51^. Previous studies have also shown that several viruses can induce autophagy, including Rift Valley fever virus (RVFV), Hepatitis B virus, Epstein Barr virus, Sindbis virus and hepatitis C virus^31,50,52–59^. In this study, we found that the LC3-II level accumulated upon SFTSV infection, which indicates that SFTSV infection may promote LC3-II formation or inhibit the autophagic degradation. While the use of autophagy inhibitors pepstatin A and E64d, which can inhibit autophagic degradation^40^, did not lead to further increase of LC3-II in SFTSV-infected cells. This means that the inhibition of autophagic degradation has already existed in SFTSV-infected cells and that the inhibition may be the main reason of un-degraded LC3-II accumulation in SFTSV-infected cells.

Many viruses, especially RNA viruses, have been found to be able to induce the formation of LC3 protein positive double-membrane vesicles (DMVs). Thus these kinds of vesicles are also called the autophagosome-like DMVs^36^. This structure has been found in the poliovirus, CVB3, and enterovirus 71^26,37,38^. Cells infected with these viruses contain accumulated autophagosome-like DMVs, which are not only LC3 positive but also morphologically similar^29^. In this study we did not find obvious differences of LC3 puncta (which is classic for autophagosomes) between SFTSV-infected cells and mock-infected cells, however, we found special LC3-positive structures in infected cells that were different from the autophagosome puncta, while none of these kinds of structures in mock-infected cells was observed. Combined with the un-degraded LC3-II accumulation detected in infected cells, we speculated that the LC3-positive structures may be LC3-II-positive structures and that SFTSV inhibited autophagic degradation to help form these structures for virus replication. further research needs to be done on how LC3-II protein participates in formation of the structures.

A previous study demonstrated that the NSs protein of SFTSV can form VLSs in infected cells^11^, this reminds us that these LC3-positive structures and the VLSs should be the same structures. Thus we detected co-localization between NSs protein and endogenous LC3 protein and found that the LC3-positive structures and NSs-induced VLSs are the same structures in SFTSV-infected cells, which indicated that these structures found in the infected cells might also be the autophagosome-like DMVs as found in several other kinds of viruses^26,37,38^.

A previous study has shown that SFTSV NSs interacts and co-localizes with RIG-I, the E3 ubiquitin ligase TRIM25, and TANK-binding kinase 1 (TBK1) into SFTSV NSs-induced cytoplasmic structures^19^. We want to know whether NSs could also co-localize with LC3 protein. We transfected the NSs-GFP plasmid into Vero cells and did not find co-localization of NSs-GFP and endogenous LC3, which indicated NSs alone could not co-localize with the LC3 protein. Thus co-localization of NSs and LC3 may need help of other SFTSV proteins expressed during SFTSV infection. However, exactly which SFTSV protein plays this role needs further study.

Viroplasm or viral replication factories are unique structures generated as a platform for efficient viral replication^60^. Viral factories for both DNA and RNA viruses have been reported in several virus families^60,61^. Previous studies have demonstrated that the viroplasm-like structures (VLS) induced by NSs protein of SFTSV is similar to the viroplasm in cells infected with rotavirus or other reoviruses and co-localizes with viral dsRNAs in SFTSV-infected cells, suggesting this NSs-formed VLS is implicated in the replication of SFTSV^11,62^. As it co-localizes with NSs protein, we wondered whether LC3 also has an influence on SFTSV replication. We transfected si-RNA of *LC3B* into Vero cells and found that knockdown of *LC3B* led to an increase in SFTSV RNA level compared to that of control group, which indicated its negative effect on SFTSV replication. However, further research is needed to determine whether LC3B protein’s inhibition of SFTSV replication occurs on these NSs-induced VLSs and the concrete mechanism of LC3’s inhibition on SFTSV replication.

Besides classical autophagy, selective autophagy plays a crucial role in the elimination of pathogenic bacteria and viruses, termed xenophagy^63^, in which autophagy adaptors including p62, NBR1 (neighbor of BRCA1 gene 1), NDP52 (nuclear dot protein 52 kDa) and optineurin are considered as PRRs that can selectively target a variety of pathogens for autophagic degradation^30^. For example, autophagic degradation of Sindbis virus involves the selective degradation of the viral capsid in a p62-dependent manner^31^. We found that NSs co-localized with p62, which indicates the possibility of selective autophagy, or xenophagy, might occur during SFTSV infection. Furthermore, we found that NSs co-localized with Lamp2b instead of Lamp1 protein. Lamp1 and Lamp2b are both lysosome markers while Lamp 2b is a crucial component for chaperone-mediated autophagy (CMA)^43^, so there may also exist the possibility of CMA taking roles in SFTSV infection. These results indicated that SFTSV infection has a close relationship with autophagy. However, NSs protein alone cannot co-localize with p62 or Lamp2b. Thus the co-localizations between NSs and LC3, p62 and Lamp2b are caused by SFTSV infection while NSs protein alone could not lead to their sequestration.

To our knowledge, we are the first to show the interaction between SFTSV and autophagy. SFTSV can promote LC3-II accumulation while LC3B protein plays a negative role in SFTSV replication. Furthermore, the NSs protein of SFTSV can co-localize with autophagy-related proteins LC3, p62 and Lamp2b in the VLS respectively during infection. However, further research needs to be done on how SFTSV proteins interact with autophagy proteins during infection. Our results give insight into how SFTSV targets autophagy pathways in host cells, which may provide a theoretical basis for prevention and treatment of SFTSV infection.

## Materials and Methods

### Cells

Vero cells (African green monkey kidney cells) were grown in Dulbecco’s modified Eagle’s medium (DMEM) (HyClone, SH30243.01) supplemented with 10% fetal bovine serum (FBS) (HyClone, SH30070).

### Reagents, antibodies and plasmids

Antibodies including anti-LC3B (ab48394), anti-SQSTM1 (ab109012), anti-EEA1 (ab2900), anti-Rab5 (ab18211), anti-Rab11a (ab65200), anti-Lamp1 (ab24170), anti-Lamp 2b(ab18529) were purchased from Abcam, GAPDH antibody (60004-1-Ig) was purchased from Proteintech, anti-NSs antibody was generated in our laboratory. E64d (E8640) and pepstatin A (P5318) were purchased from Sigma Aldrich. Conjugated antibodies including the IRDye 800CW goat anti-mouse IgG (926-32210) and IRDye 800CW anti-rabbit IgG (926–32211) were purchased from LI-COR. Fluorescent secondary antibodies including Alexa Fluor 488-conjugated anti-mouse (SA00006-1) and anti-rabbit IgG (SA00006-2), Alexa Fluor 594-conjugated anti-mouse (SA00006-3) and anti-rabbit IgG (SA00006-4) were purchased from Proteintech. DAPI reagent (C0060) was purchased from Solarbio. EGFP-LC3B (11546, deposited by Toren Finkel) was purchased from Addgene. NS-GFP vector was re-constructed by cloning the SFTSV-NSs open reading frame into the pEGFP-N1 vector (preserved in our laboratory). Both siRNA directed against *LC3B* (GCUUACAGCUCAAUGCUAA) and negative control RNA was synthesized by GenePharma.

### Quantitative real-time PCR

Vero cells were transfected with negative control RNA or *LC3B* silence-RNA for 24 h before being infected with SFTSV (MOI = 0.01), cells and supernatant were harvest at 24 h, 48 h and 72 h, respectively for RNA extraction. Total RNA was extracted from cells and supernatant respectively by using RNeasy MINI Kit (QIAGEN, Cat No. 74106) and QIAamp Viral RNA Mini Kit (QIAGEN, Cat No. 52906). Extracted RNA was used for reverse transcription with Reverse Transcription System (PROMEGA, A3500). Then cDNA was used for Real time-PCR detection using the Lightcycler 480 SYBR Green (Roche, Cat. 04887352001). The primers used were forward primer (5′-GGGTCCCTGAAGGAGTTGTAAA-3′) and reverse primer (5′-TGCCTTCACCAAGACTATCAATGT-3) for the partial L segment of SFTSV and forward primer (5′-CGAGAGCAGCATCCAACCAA-3′) and reverse primer (5′-CACCAACAGGAAGAAGGCTTGA-3) for LC3 detection.

### Cell treatment and virus infection

Cells were mock-infected or SFTSV-infected, treated with inhibitors, transfected with plasmids or siRNA and then processed for Western blot, immunofluorescence staining or RNA extraction. For the infection experiment, SFTSV was added to cells at MOI = 0.01 for the *LC3B* silencing experiment and at MOI = 1 to 5 for the rest of the experiments. Inhibitors were added to the culture medium 12 h after SFTS phlebovirus infection. E64d and pepstatin A were added to the culture medium simultaneously, both with concentration of 10 μM; a certain amount of methanol and acetic acid were added to the control group of cells. For the transfection experiment, cells were grown to 60% to 70% confluence on coverslips, 6-well plates or 24-well plates and transfected with plasmids by using Lipofectamine-2000 reagent (Life Technologies, Cat.11668019) according to manufacturer’s recommendations. Six hours after transfection, culture medium was changed to complete medium and cells were managed with different kinds of treatment.

### Western blot

After certain treatment, cells were washed with PBS and lysed with RIPA Lysis Buffer (Beyotime, P0013B) containing 1% Triton X-100 in 50 mM TRIS-HCl, pH 7.4, 150 mM NaCl and PMSF (Beyotime, ST506). The lysate was centrifuged at 13,000 × g for 10 min at 4 °C and then heated at 100 °C for 5 min. Equal amounts of protein extracts were separated on 15% polyacrylamide gels and transferred onto 0.20 μm PVDF membranes (ISEQ. 00010, Merck). Membranes were blocked in 5% skim milk (232100, BD) dissolved in TBS (Tris-base 1.5 mM, Tris-HCl 8.5 mM, NaCl 150 mM) for 1 h at room temperature. Membranes were then incubated with primary antibodies and secondary antibodies diluted in TBST (1*TBS, 0.1% Tween-20) orderly. Membranes were visualized using Odyssey infrared imaging system (School of Public health, Shandong University, Jinan, China) from LI-COR. Band intensities were quantified with ImageJ software.

### Immunofluorescence staining and confocal microscopy

Cells were seeded on coverslips (801007, NEST) in a 24-well plate. The infected cells were fixed and permeabilized in Immunol Staining Fix Solution (P0098, Beyotime) for 10 min at room temperature; blocked with Immunol Staining Blocking Buffer (P0102, Beyotime) for 1 h prior to incubation with primary antibodies diluted in PBS followed by incubation with Alexa 488/549 coupled appropriate secondary antibody diluted in PBS. Cell nuclei were stained with DAPI, and the coverslips were mounted onto glass slides by using 50% glycerin diluted by PBS. Laser-scanning confocal microscopy was performed on a Zeiss LSM780 (Public Technology Experimentation Center, Shandong University, Jinan, China). All images were taken with the same acquisition condition, brightness/contrast of images adjusted only for presentation purposes.

### Anti-NSs antibody

The NSs fragment of SFTSV was cloned into the PVAX-1 vector. The PVAX-NSs vector was then injected into the BABL/c mouse by multi-site intramuscular injection. The sera of mice were obtained 30 days after injection. The anti-NSs specificity of the sera was confirmed by Western blot analysis of PVAX-NSs-transfected Vero cells, PVAX-transfected cells and SFTSV-infected cells. The anti-NSs serum reacted with both PVAX-NSs-transfected Vero cells and SFTSV-infected cells but not with PVAX-transfected cells. ELISA was used to detect serum titer by covering the board with SFTSV suspension.

### Ethics Statement

This study was approved by the ethics committee of Shandong University. All procedures were carried out in accordance with the guidelines for animal care published by the United States’ National Institutes of Health (NIH) for animal care (Guide for the Care and Use of Laboratory Animals).

### Statistical analysis

Statistical evaluations were performed using the Student t test. Differences were considered significant at values of P < 0.05; 0.01–0.05 was considered significant (*); < 0.01 was considered very significant (**).

### Data availability

The datasets generated and analyzed during the current study are available from the corresponding author on reasonable request.
